# Activation of transcription factor AP-1 in response to thermal injury in rat small intestine and IEC-6 cells

**DOI:** 10.1186/s12876-015-0309-z

**Published:** 2015-07-11

**Authors:** Yonghong Zhang, Hong Zhao, Tao Liu, Changrong Wan, Xiaoxi Liu, Zhimin Gao, Xiaolin Hou, Linshu Jiang, Fenghua Liu

**Affiliations:** 1 Beijing Key Laboratory for Dairy Cow Nutrition, Beijing University of Agriculture, No. 7, Beinong Road, Changping District, Beijing, 102206 P. R. China; 2 TCVM Laboratory, College of Veterinary Medicine, China Agricultural University, Beijing, 100193 China

**Keywords:** AP-1, NF-κB, Heat stress, Rat small intestine, IEC-6

## Abstract

**Background:**

Our previous studies indicated that heat stress can cause significant damage to the intestinal epithelium and induce differential expression of many genes in rat small intestine. The transcription factors AP-1 and NF-κB, which act as important mediators by binding to specific DNA sequences within gene promoters, regulate the transcription of genes associated with immune regulation, stress response and cell fate.

**Methods:**

To determine whether AP-1 and NF-κB are involved in hyperthermia-induced injury in rat small intestine and IEC-6 cells, we investigated their activity, and the expression of related proteins, by electrophoretic mobility shift assays and western blotting, respectively.

**Results:**

Heat stress resulted in severe damage to the epithelium of the small intestine. The cell morphology and viability were obviously altered when IEC-6 cell was exposed to hyperthermia. AP-1 was activated in the small intestine of heat-stressed rats, as was phosphorylation of the JNK signaling pathway. In IEC-6 cell line, AP-1 activation in groups exposed to 42 °C for 1 h, 2 h and 4 h was significantly increased. In contrast, NF-κB was not activated in both *in vivo* and *in vitro* models.

**Conclusion:**

These results reveal that AP-1 is likely to play an important role in regulating gene transcription in rat small intestine and IEC-6 cells during exposure to heat stress.

**Electronic supplementary material:**

The online version of this article (doi:10.1186/s12876-015-0309-z) contains supplementary material, which is available to authorized users.

## Background

As one important physical stimulus, ambient temperature can evoke a series of drastic changes in biological function [1] including gastrointestinal injury and dysfunction [2]. Our previous studies have shown that heat stress can induce damage in the rat small intestine, along with differential expression of many genes associated with immune regulation and metabolism, and those encoding regulatory peptides [3]. A number of growth-related molecules (such as Gdf15, Gdf9, Ctgf, and Egfr) which are critical for cellular survival, proliferation and migration, have also been shown to be differentially expressed in response to hyperthermia-induced damage [4].

Transcription factors, important mediators involved in signal transduction, bind to specific DNA sequences within gene promoters, and thus regulate transcriptional activity. Both NF-κB and AP-1 are well known pleiotropic transcription factors that independently and/or complementarily regulate a large number of genes related to a wide range of functions, including immune regulation, proliferation, differentiation, and apoptosis [5, 6].

NF-κB is a ubiquitous transcription factor and a member of a family of proteins that are important regulators of a variety of responses. NF-κB exists as a dimer predominantly composed of p50 and p65 subunits, although it also contains other family members, such as RelB, c-Rel, v-Rel and p52 [7]. The activity of NF-κB is regulated by a family of IκB inhibitor proteins [8], which sequester NF-κB in the cytoplasm. In response to various external pathogenic stimuli, IκB is phosphorylated, ubiquitinated, and subsequently degraded by a proteosome-dependent pathway. Degradation of IκB allows NF-κB to translocate into the nucleus, where it binds to specific promoter elements and induces gene transcription.

AP-1 is a central switch to convert extracellular signals into genetic responses and to determine cell proliferation, differentiation, and apoptosis. AP-1 complex consists of homodimers and heterodimers formed by a group of transcription factors, including members of the Jun, Fos, and ATF families [9]. Previous studies indicate that the c-Jun/ATF-2 heterodimer is one of the main components of expression pathways associated with oncogenesis [9] and the extreme cellular stress of ischemia and reperfusion [10]. JNK is one member of the mitogen-activated protein kinase (MAPK) family, which play crucial roles in many responses [11]. JNK was initially described as a stress-induced protein kinase acting to phosphorylate the NH2-terminus of the transcription factor c-Jun; hence, this pathway is often referred to as the stress-activated protein kinase (SAPK) pathway [12]. Multiple stresses increase JNK activity including UV, r-irradiation, cytotoxic drugs, ischemia and reactive oxygen species. JNK phosphorylates several transcription factors including c-Jun, ATF-2, and p53 [13], which in turn regulate the expression of genes mediating cell proliferation, differentiation or apoptosis. Many studies have shown that there is crosstalk between JNK1 and NF-κB [14].

To further explore the mechanism of gene expression involved in hyperthermia-induced damage and repair in the rat small intestine, we investigated the activity of transcription factors AP-1and NF-κB and determined the expression of proteins acting upstream in their respective pathways, using both *in vivo* and *in vitro* models.

## Methods

### Animals and treatments

All protocols and procedures involving animals were approved by the Beijing University of Agriculture Institutional Animal Care and Use Committee, and conducted in accordance with the committee’s guidelines. 12 male Sprague–Dawley (SD) rats weighing 200 ± 20 g (obtained from Beijing Vital River Laboratory, Animal Technology Co., Beijing, China) were caged at 25 °C, with a 12 h light:dark cycle and free access to food and water for 7 days. Rats were then randomly divided into control or heat-stress groups (6 rats per group) and housed in an artificial climate chamber (HPG-400BX, Harbin Donglian Electronic Technology, Heilongjiang, China) under normal conditions (25 °C, 60 % relative humidity). Rats in the heat treatment group were exposed to 40 °C and 60 % RH from 11:00 to 13:00 for 3 consecutive days. The detail of heat-stress procedure was previously described by Yu *et al.* [4]. Rat rectal temperature was recorded daily before and after heat treatment using a thermistor probe connected to a digital thermometer. Body weight was recorded daily during the 7 adaptation days and the 3 treatment days.

### Sampling

All rats were euthanized by decapitation without anesthesia immediately after the final 2 h heat treatment period. After euthanasia, trunk blood was collected and centrifuged at 3,000 × g for 10 min and the sera stored at −20 °C until required. Sections of the duodenum, jejunum and ileum were rapidly excised and washed with physiological saline. All intestinal segments were divided into two parts: 1) a section of 1 cm length was fixed in 10 % neutral formalin for paraffin embedding; 2) a section of 3 cm length was minced and separated into three sample tubes, snap frozen in liquid nitrogen and stored at −80 °C until required.

### Serum cortisol analysis and morphological examination

Serum cortisol concentration was determined using an I^125^ cortisol radioimmunoassay kit, according to the manufacturer’s instructions (Beijing Chemclin Biotech Co., Ltd, China). Formalin-fixed samples were embedded in paraffin and sectioned (5 μm thick) in transverse orientation. After deparaffinization and dehydration, sections from the duodenum, jejunum and ileum were stained with hematoxylin and eosin. The structure of the mucosa was observed using an Olympus BH2 microscope (Olympus, Tokyo, Japan).

### Cell culture and treatments

Rat IEC-6 cells (#CRL21592, purchased from Peking Union Medical College, Beijing, China) were cultured in Dulbecco’s modified Eagle medium containing 5 % (v/v) fetal bovine serum (HyClone, Logan, UT, USA), 2 mg/l insulin, 50 IU/ml penicillin and 50 μg/ml streptomycin and incubated at 37 °C under 5 % (v/v) CO2. The medium was replaced 24 h following initial cell plating. Control group cells were kept strictly at 37 °C under 5 % CO2, while cells of the heat treatment groups were exposed to 42 °C under 5 % CO2 in the incubator (Thermo, Marietta, Ohio, USA) for 15 min, 30 min 1 h, 2 h, 4 h, and 8 h, respectively. To inhibit specific intracellular agents, cells were pretreated with 10 μM SP600125 (JNK inhibitor, #1496, Tocris Bioscience, Bristol, UK) for 1 h prior to heat treatment. Changes in cell morphology following all treatments were observed using a phase-contrast inverted biological microscope (IX71/IX2, Olympus).

### MTT cell viability assay

To measure cell viability, equivalent numbers of IEC-6 cells were plated on 96-well multiplates and cultured in DMEM containing 5 % fetal bovine serum at a density of 1.2 × 10^5^ cells /ml. After the cells were attached to the multiplate, control cells were maintained at 37 °C, while heat-stressed cells were submitted to 42 °C for 15 min, 30 min, 1 h, 2 h, 4 h, and 8 h, respectively. Following the heat stress period, 10 μl of MTT (10 mg/ml) was added to each well then incubated at 37 °C for 4 h. Well media was aspirated and the formazan product dissolved using dimethyl sulphoxide. The remaining formazan product was analysed using a microplate reader (BIO-RAD, USA) at a fixed absorption wavelength of 570 nm. Survival rates were calculated as percent OD570 of the untreated cells normalized to the ‘zero’ survival value.

### Protein extraction and measurement

Nuclear and cytoplasmic extracts from rat small intestine and IEC-6 cells were prepared using the Nuclear and Cytoplasmic Extraction Reagent Kit (KeyGEN Biotech, Nanjing, China). Protein content was determined using the Pierce BCA protein assay kit (Thermo Fisher Scientific, Rockford, IL, USA) using bovine serum albumin as a standard.

### Electrophoretic mobility shift assay (EMSA)

The nuclear fraction was used for EMSA analysis of AP-1 and NF-κB. IRDye700-labeled AP-1 (sense: 5’-CGC TTG ATG ACT CAG CCG GAA-3’; antisense: 5’-TTC CGG CTG AGT CAT CAA GCG-3’) and NF-κB (sense: 5’-AGT TGA GGG GAC TTT CCC AGG C-3’; antisense: 5’-GCC TGG GAA AGT CCC CTC AAC T-3’) oligonucleotides were purchased from LI-COR Biosciences (Lincoln, NE, USA). Briefly, EMSA binding reactions were performed by incubating 2 μg of nuclear extract with the annealed oligonucleotides and binding reagents for 30 min at room temperature in the dark. For the supershift assay, antibodies (Cell Signaling Technology, Inc., Danvers, MA, USA) were incubated with samples after the initial binding reaction between nuclear proteins and the oligonucleotides. The reaction mixture was subjected to electrophoresis on a 5 % native gel at 4 °C in the dark. The gel was scanned using an Odyssey Infrared Imaging System (LI-COR Biosciences).

### Western blotting

Twenty μg of either cytoplasmic or nuclear lysate were resolved on a 12 % polyacrylamide-sodium lauryl sulfate gel via electrophoresis and transferred to nitrocellulose membranes. After blocking, membranes were incubated overnight at 4 °C with respective primary antibodies (Cell Signaling Technology, Inc.) diluted to 1:1000. The blots were then incubated with a 1:15000 dilution of the anti-rabbit secondary antibody labeled with IRDye700 (LI-COR Biosciences) at room temperature for approximately 1–2 h. Finally, the membranes were scanned using an Odyssey Infrared Imaging System (LI-COR Biosciences).

### Statistical analysis

Data analyses were carried out using SPSS12.0 (SPSS Inc., USA) and graphs were created using Origin6.0 (OriginLab, Northampton, USA). Differences between two groups were assessed using Student’s *t*-test, or for more than two groups using an analysis of variance (ANOVA) combined with the *post-hoc* LSD test. Data are expressed as mean ± S.E. Differences were considered statistically significant at p < 0.05.

## Results and discussion

### Assessment of heat treatment in the animal model

**Fig. 1:**
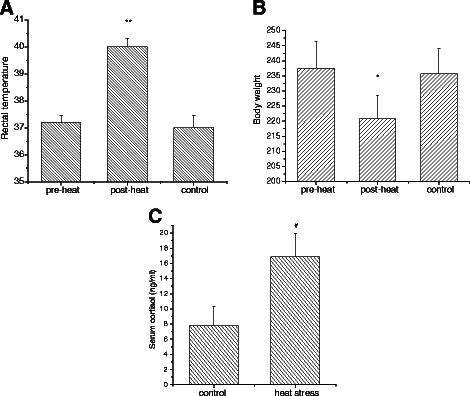
Assessment of hyperthermia in an animal model. (**a**) Rat rectal temperature before and after heat treatment. Rat rectal temperatures were significantly elevated following 2 h heat exposure at 40 °C. (**b**) Rat body weight before and after heat stress. Rat body weight was significantly decreased after heat treatment. (**c**) Mean cortisol concentration in the heat-stressed group was significantly higher than that of the control group. Values represent the mean ± S.E. for n = 6 rats per group. *p < 0.05, **p < 0.01 indicate a significant difference for the rats before and after heat stress. #*p* < 0.01 indicates that the heat-stress group was significantly different from the control group

In mammals, rectal temperature, serum cortisol level and body weight are used for a basic assessment of heat stress [15]. Fig. 1 summarizes these main characteristics. Compared with the results before heat stress, rectal temperature was significantly increased after treatment. Rat body weight was significantly decreased after heat treatment (220.8 ± 8.5 g) compared with before treatment (237.5 ± 7.8 g). There were no significant differences in either rectal temperature or body weight between the control group and the heat-stress group before treatment. Measurement of serum hormones showed that heat stress induced a significant increase in cortisol concentration compared with the control rats. These results are consistent with our previous reports [4, 16], in which the same model was used.

### Histological analysis

**Fig. 2:**
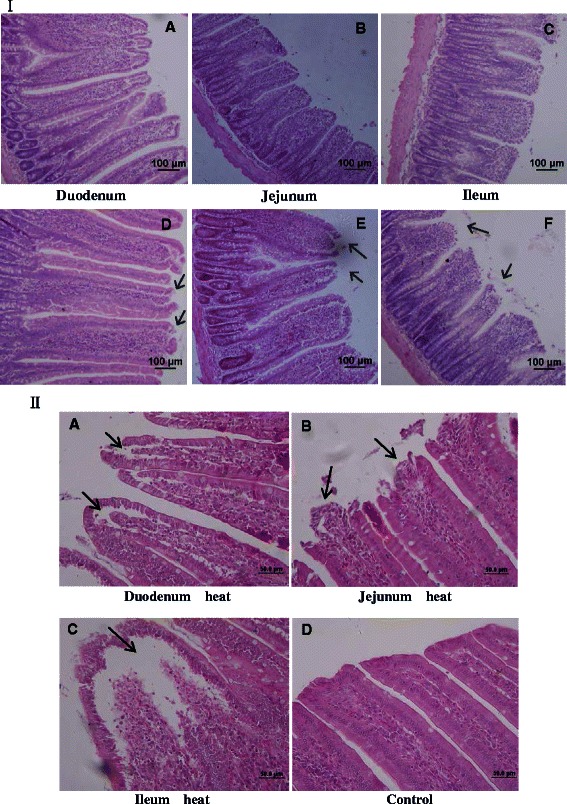
Morphology of rat small intestine in response to heat stress. (**I**) Photomicrographs of hematoxylin and eosin-stained sections of rat small intestine after 3 days of heat treatment. Upper panels show small intestine from control rats, lower panels show small intestine from heat-stressed rats. Thermal injury to the intestinal villi is apparent, with hyperemia desquamation at the tips of the intestinal villi. (**II**) Morphology of rat small intestine at higher magnification. Heat stress resulted in profound damage to the epithelium of the small intestine. Sloughing of epithelium off the basement membrane at the villus tips and even exposing of the lamina propria are more obvious at higher magnification. Abnormal microstructures are indicated by arrows

Many environmental and biological stressors including radiation, hyperthermia (heat stress), LPS, various drugs, endotoxins, and ROS can cause significant damage to the intestinal epithelium [17]. In the case of hyperthermia, significant injury to the intestine is sustained because of reduced blood flow to the gut [2], resulting in ischemia within the small intestine [18]. Light micrographs of hematoxylin and eosin-stained small intestine tissue demonstrated that heat stress resulted in profound damage to the epithelium of the small intestine (Fig. 2I). Sloughing of epithelium off the basement membrane at the villus tips was observed in the heat-stressed tissue compared with the control tissue. Vacuolization of epithelial cells was also observed at higher magnification and, in severe cases, the lamina propria was exposed (Fig. 2II).

### Cell morphology and viability

**Fig. 3:**
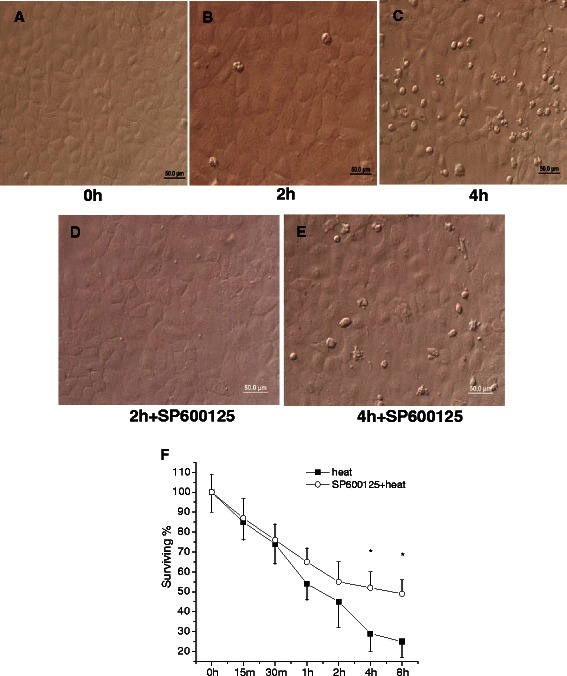
Morphology and viability of IEC-6 cells in response to heat stress. Normal IEC-6 cells in control group (**a**). Cellular morphology was markedly altered following heat treatment (42 °C for 4 h), with changes in cell shape and a greater number of dead cells in the supernatant (**b** and **c**). This effect was attenuated by pretreating cells with JNK inhibitor SP600125 (**d** and **e**). IEC-6 cell viability gradually decreased over time and SP600125 had a positive effect on cell viability (**f**). It was measured by MTT assay, in which IEC-6 survival rate was calculated as percent OD570 of the untreated group (0 h). Data are mean ± S.E., n = 3 per treatment. * p < 0.05, compared to respective heat-stress group

A previous report [19] indicated that the effect of heat treatment on IEC-6 cells was dependent on temperature and exposure time. According to methods used in our own previous study [4], IEC-6 cells were subjected to heat stress of 42 °C. Following heat exposure, cells were examined under a phase-contrast inverted microscope. The morphology was markedly altered, with a different cell shape, and a greater dead cell mass was clearly observed in the supernatant after 4 h (Fig. 3C) of the cells being exposed to heat. In contrast, the damage was attenuated with addition of JNK inhibitor SP600125 (Fig. 3d and e). Compared to control cells, IEC-6 demonstrated 70 % reduction of viability after 4 h at 42 °C, whereas cells pretreated with SP600125 were relatively resistant to the toxic effect of heat (Fig. 3f).

### Effects of heat stress on AP-1 and JNK pathway

**Fig. 4:**
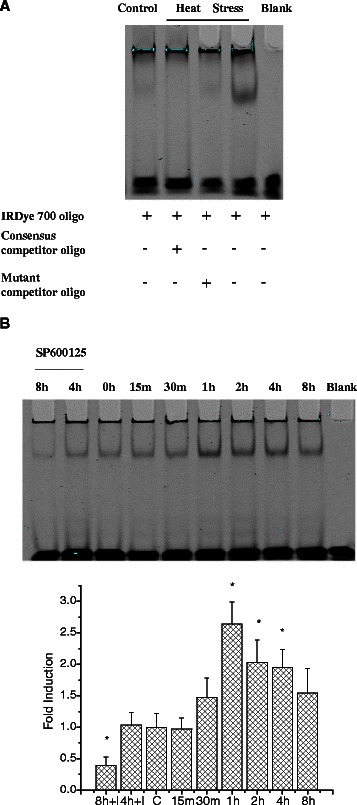
Determination of AP-1 using electrophoretic mobility shift assay (EMSA). (**a**) AP-1 was activated in rat small intestine after heat stress (lane4). (**b**) The kinetic profile of AP-1 activity in response to heat stress in IEC-6 cells. Peak AP-1 activation occurred at 1 h. Data are mean ± S.E., n = 3 per treatment. *p < 0.05, compared to 0 h

EMSA assessment of AP-1 activation was performed in rat small intestine and IEC-6 cells. Fig. 4 shows the results of representative experiments *in vivo* and *in vitro*. Compared with the control group, the activity of AP-1 in heat-stressed rats was increased (Fig. 4a). Following heat exposure, IEC-6 cells were harvested at time 0 h, then at 15 min, 30 min, 1 h, 2 h, 4 h, and 8 h. AP-1 activation in groups treated for 1 h, 2 h and 4 h was significantly increased (P < 0.05) compared to 0 h (Fig. 4b).

**Fig. 5:**
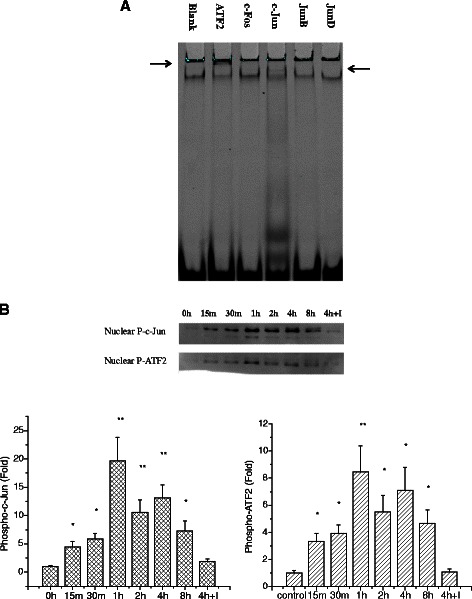
(**a**) EMSA Supershift was conducted to identify the contributing family members. Lane 2 and lane 4 showed that the AP-1 complex might be composed of ATF2 and c-Jun (indicated by arrows). Western blotting was performed to identify the specific members (**b**). Both c-Jun and ATF2 were activated in heat-stress groups and got their highest phosphorylation level after 1 h treatment. Data are mean ± S.E., n = 6 per treatment. *p < 0.05, compared to control group, **p < 0.01, compared to control group. 4 h + I: cells pretreated with JNK inhibitor SP600125 and exposed to heat for 4 h

To investigate the specificity of the AP-1 complex, supershift experiments were carried out by adding antibodies against c-Jun, JunB, JunD, c-Fos and ATF2 to the nuclear extracts from IEC-6 cells exposed heat for 1 h. Protein-antibody recognition can be visualized by a decrease in the mobility of the DNA-protein complex and a diminution of the AP-1 complex. Fig. 5a implies that the AP-1 complex is likely to consist of c-Jun and ATF2. However, the hysteretic bands were too vague to confirm this postulation and we could not get better results. Then, western blot analysis was conducted to identify the contributing family members. We found that heat stress did induce the phosphorylation of c-Jun and ATF2 (Fig. 5b). What’s more, both c-Jun and ATF2 followed similar kinetic profiles compared with that observed for AP-1(Fig. 4b). Taparowsky *et al.* found that ATF2 and c-Jun mutually regulate each other to function in the stress response mainly through altering the dynamics of subcellular localization and positively impacting transcriptional activity [20].

**Fig. 6:**
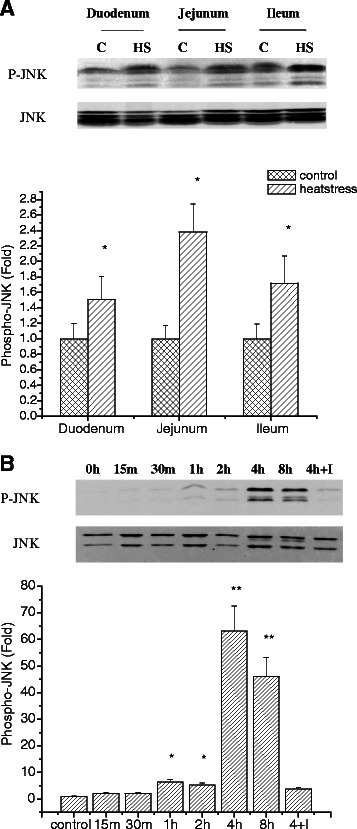
Western blotting was performed to detect (**a**) phosphorylation levels of JNK in three segments of small intestine, (**b**) the kinetic profile of JNK phosphorylation in IEC-6 cell line in response to hyperthermia. *In vivo*, heat stress significantly increased JNK phosphorylation compared to the control groups. *In vitro*, JNK phosphorylation was maximum at 4 h and reversible by the addition of JNK inhibitor. Data are mean ± S.E., n = 6 per treatment. *p < 0.05, compared to control group, **p < 0.01, compared to control group. C: control; HS: heat stress; 4 h + I: cells pretreated with JNK inhibitor SP600125 and exposed to heat for 4 h

The involvement of AP-1 activation in our heat-stress models prompted us to ask whether the JNK pathway might also be involved. To test this hypothesis, western blot analysis of JNK phosphorylation was performed using the cytoplasmic extract from rat small intestine and IEC-6 cells. In all three segments of small intestine, heat stress was found to significantly increase JNK phosphorylation in contrast with the control group (Fig. 6a). In IEC-6 cells, JNK phosphorylation was also increased. This effect reached a maximum at 4 h and lasted for several hours, and was reversible by the addition of JNK inhibitor SP600125 (Fig. 6b). As the main protein upstream of AP-1, JNK is activated first, leading to the phosphorylation of pre-existing c-Jun and ATF2 proteins [21]. To investigate whether JNK inhibition influences the activation of AP-1 during heat stress, IEC-6 cells were subjected to heat combined with SP600125. Fig. 4b and Fig. 5b show that AP-1 activity is significantly attenuated by the JNK inhibitor SP600125.

### Assessment of NF-κB by EMSA and Western blot

It is widely known that rather than being a mediator of the immune response, NF-κB more generally represents a regulator of stress responses [22]. NF-κB activity can be induced by various stressors including hyperglycemia, hyperthermia, hyperosmotic shock, reactive oxygen species, ischemia/reperfusion, and irradiation. Electrophoretic mobility shift assays were conducted towards transcription factor NF-κB. To our surprise, NF-κB activation in both rats and IEC-6 cells was not observed (Additional file 1: Figure S1). To confirm this result, we investigated main proteins related to the NF-κB signaling pathway by western-blot. Exposing rats and IEC-6 cells to hyperthermia did not induce significant phosphorylation of P50 and P65, compared with respective control groups (Additional file 1: Figure S2). Our result was not consistent with previous report [23], in which hyperthermia enhanced the transcriptional activity of both AP-1 and NF-κB in PMA/ionomycin treated T cells. Although Ap-1 activity was more rubost in this model, NF-κB was obviously activated in the presence of heat stress. It’s not clear why there was a lack of NF-κB activation in our models. It may be related to the specificity of both stimulus and cell type. Karin had pointed out that not all cell types responded equally to a given stimulus, and that not every stimulus could activate NF-κB in every cell type examined [24]. On the other hand, it’s reported that thermal stress could alter metabolic processes impacting upon intracellular oxidation-reduction status and thereby inhibit the activity of NF-κB [25]. Our published microarray analysis data had shown that many genes related to oxidation-reduction and metabolism pathways did differently express in response to heat stress [3].

In summary, we have demonstrated that the transcription factor AP-1 (primarily its c-Jun and ATF-2 components) can be activated in rat small intestine and IEC-6 cells exposed to heat stress, and that the JNK signaling pathway is also involved in this response. However, it remains to be established whether activation of c-Jun/ATF2 leads to an enhanced level or activity of the genes identified by DNA microarray. Also, the mechanism of NF-κB inactivation needs more in-depth investigations.

## Conclusion

Ap-1 was activated in rat small intestine and IEC-6 cells when exposed to heat stress. It is likely to play an important role in regulating gene transcription in these models.

## Additional file

Additional file 1: Figure S1. Electrophoretic mobility shift assay for NF-κB *in vivo* (A) and *in vitro* (B). NF-κB activation was not observed in both rat small intestine and IEC-6 cells. **Figure S2**. Western blotting was performed to detect nuclear p65 and p50 phosphorylation levels *in vivo* (A) and *in vitro* (B). There were no significant differences. Data are mean ± S.E., n = 6 per treatment. C: control; HS: heat stress.
