# Supplementary material for: Activation of transcription factor AP-1 in response to thermal injury in rat small intestine and IEC-6 cells
Source: BMC Gastroenterol. 2015 Jul 11;15:83. doi: 10.1186/s12876-015-0309-z (PMC4498520; doi:10.1186/s12876-015-0309-z)
Supplement: Additional file 1: Figure S1. — Electrophoretic mobility shift assay for NF-κB in vivo (A) and in vitro (B). NF-κB activation was not observed in both rat small intestine and IEC-6 cells. Figure S2. Western blotting was performed to detect nuclear p65 and p50 phosphorylation levels in vivo (A) and in vitro (B). There were no significant differences. Data are mean ± S.E., n = 6 per treatment. C: control; HS: heat stress. [file 12876_2015_309_MOESM1_ESM.docx]

**A**

| + | + | + | + | + |
| --- | --- | --- | --- | --- |
| - | + | - | - | - |
| - | - | + | - | - |

**IRDye 700 oligo**

**Consensus**

**competitor oligo**

**Mutant**

**competitor oligo**

**Control Heat Stress Blank**


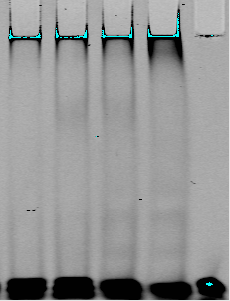


**B**

**SP600125**


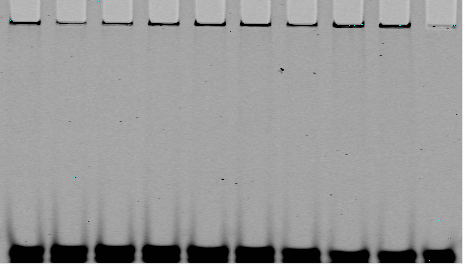


**8h 4h 0h 15m 30m 1h 2h 4h 8h Blank**

Figure S1. Electrophoretic mobility shift assay for NF-κB in vivo (A) and in vitro (B). NF-κB activation was not observed in both rat small intestine and IEC-6 cells.

**0h 15m 30m 1h 2h 4h 8h**

Nuclear P-P50


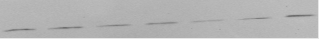

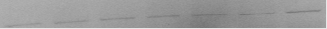


Nuclear P-P65

**A**

**C HS C HS C HS**

Nuclear P-P50


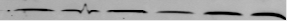

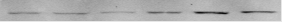


Nuclear P-P65

**Ileum**

**Duodenum**

**Jejunum**

**B**

Figure S2. Western blotting was performed to detect nuclear p65 and p50 phosphorylation levels in vivo (A) and in vitro (B). There were no significant differences. Data are mean ± S.E., n=6 per treatment. C: control; HS: heat stress.
